# Assessment of ibrutinib plus rituximab in front-line CLL (FLAIR trial): study protocol for a phase III randomised controlled trial

**DOI:** 10.1186/s13063-017-2138-6

**Published:** 2017-08-22

**Authors:** Laura Collett, Dena R. Howard, Talha Munir, Lucy McParland, Jamie B. Oughton, Andy C. Rawstron, Anna Hockaday, Claire Dimbleby, David Phillips, Kathryn McMahon, Claire Hulme, David Allsup, Adrian Bloor, Peter Hillmen

**Affiliations:** 1 Clinical Trials Research Unit, Leeds Institute of Clinical Trials Research, University of Leeds, Leeds, UK; 2 St James’ Institute of Oncology, St James’ University Hospital, Leeds, UK; 3 Academic Unit of Health Economics, Leeds Institute of Health Sciences, University of Leeds, Leeds, UK; 4 Haematological Malignancy Diagnostic Service, St James’ Institute of Oncology, St James’ University Hospital, Leeds, UK; 5 Hull York Medical School, Department of Haematology, Queens Centre for Oncology and Haematology, Castle Hill Hospital, Hull and East Yorkshire Hospitals NHS Trust, Cottingham, UK; 6 Department of Haematology, The Christie NHS Foundation Trust, University of Manchester, Manchester Academic Health Sciences Centre, Manchester, UK

**Keywords:** CLL, FCR, Rituximab, Ibrutinib, Front-line, Phase III, Minimal residual disease (MRD), Randomised, Clinical trial

## Abstract

**Background:**

Treatment of chronic lymphocytic leukaemia (CLL) has seen a substantial improvement over the last few years. Combination immunochemotherapy, such as fludarabine, cyclophosphamide and rituximab (FCR), is now standard first-line therapy. However, the majority of patients relapse and require further therapy, and so new, effective, targeted therapies that improve remission rates, reduce relapses, and have fewer side effects, are required. The FLAIR trial will assess whether ibrutinib plus rituximab (IR) is superior to FCR in terms of progression-free survival (PFS).

**Methods/design:**

FLAIR is a phase III, multicentre, randomised, controlled, open, parallel-group trial in patients with previously untreated CLL. A total of 754 participants will be randomised on a 1:1 basis to receive standard therapy with FCR or IR. Participants randomised to FCR will receive a maximum of six 28-day treatment cycles. Participants randomised to IR will receive six 28-day cycles of rituximab, and ibrutinib taken daily for 6 years until minimal residual disease (MRD) negativity has been recorded for the same amount of time as it took to become MRD negative, or until disease progression. The primary endpoint is PFS according to the International Workshop on CLL (IWCLL) criteria. Secondary endpoints include: overall survival; proportion of participants with undetectable MRD; response to therapy by IWCLL criteria; safety and toxicity; health-related quality of life (QoL); and cost-effectiveness.

**Discussion:**

The trial aims to provide evidence for the future first-line treatment of CLL patients by assessing whether IR is superior to FCR in terms of PFS, and whether toxicity rates are favourable.

**Trial registration:**

ISRCTN01844152. Registered on 8 August 2014, EudraCT number 2013-001944-76. Registered on 26 April 2013.

**Electronic supplementary material:**

The online version of this article (doi:10.1186/s13063-017-2138-6) contains supplementary material, which is available to authorized users.

## Background

### Chronic lymphocytic leukaemia (CLL)

Chronic lymphocytic leukaemia (CLL) is the most common adult leukaemia, affecting 7.0 per 100,000 population [1]. The incidence of CLL increases with age and twice as many men are affected as women. CLL results from the clonal proliferation of B-cells and is diagnosed by the pattern of expression of various cell surface antigens on the CLL cells. Patients most commonly present with lymphocytosis, lymphadenopathy, splenomegaly and systemic symptoms such as fatigue, weight loss and malaise. The clinical course of CLL is highly variable with a median survival from diagnosis in the region of 7 years. Patients with more advanced disease (Binet’s stages B, C and progressive stage A) have a significantly worse survival.

### Therapy for CLL

Over the last few years there has been substantial improvement in the treatment of CLL. First-line therapy has moved away from monotherapy using chlorambucil to combination immunochemotherapy such as fludarabine, cyclophosphamide and rituximab (FCR) following evidence from large randomised trials [2]. FCR is the standard therapy, shown to improve survival over comparable treatments in fit and/or young patients, but only 75% tolerate a full six cycles. In addition, there is a small risk of developing late myelodysplasia and acute leukaemia, thought to be due to the FC chemotherapy. The majority of patients relapse and require further therapy that is more difficult to administer, and associated with significant morbidity and potential mortality. Eventually the majority of patients die from CLL. Hence, more effective, targeted therapies that improve remission rates and reduce relapses, with fewer side effects, are required.

### Ibrutinib

The proliferation of CLL cells in vivo is dependent on signalling through the B-cell receptor (BCR) which is expressed at low levels on CLL cells. BCR activation leads to the transduction of signals through a series of intracellular molecules resulting in CLL cell proliferation. This provides a series of potential therapeutic targets on the BCR signalling pathway. Bruton’s tyrosine kinase (Btk) is a key component of the BCR pathway to the extent that individuals born with mutated Btk produce no B-cells. Ibrutinib [3] is an orally administered, small molecule selective irreversible inhibitor of Btk currently under investigation in B-cell malignancies including CLL and small lymphocytic lymphoma (SLL). In vitro, ibrutinib inhibits purified Btk and selected members of the kinase family with 10-fold specificity compared with off-target kinases. Ibrutinib has shown extremely promising results in relapsed, refractory CLL as well as in a small number of treatment-naïve patients [4, 5].

### Rationale for proposed study

Combination chemotherapy with FCR has been shown to be an effective treatment in patients with CLL who are able to tolerate it. However, FCR is associated with significant short- and long-term toxicity with virtually all patients relapsing and eventually becoming refractory to therapy before dying, either as a complication of the disease or its therapy (usually due to infection). Clear evidence that CLL-cell proliferation is dependent on stimulation through the B-cell receptor presents new potential targets for CLL treatment, particularly since this pathway is specific to B-cells. Several molecules on the B-cell receptor pathway have been targeted including Syk, PI3K delta and Btk, and all demonstrate activity. Of these agents it appears that ibrutinib is the most potent agent with relatively minor side effects in the early phase II trials, and the combination of ibrutinib with a monoclonal antibody should lead to a more rapid achievement of an objective response with the potential to stop ibrutinib earlier than with monotherapy. Therefore, the combination of ibrutinib and rituximab is a logical way to improve the therapy in CLL.

## Methods/design

### Trial aims and objectives

The trial aims to compare the effect on progression-free survival (PFS) of ibrutinib plus rituximab (IR) with that of FCR in young/fit patients with previously untreated CLL.

The primary objective of the trial is to assess whether IR is superior to FCR in terms of PFS, as defined by IWCLL criteria [6]. Secondary objectives include:

- Overall survival (OS)
- The proportion of participants with undetectable minimal residual disease (MRD), as defined by IWCLL criteria
- Response to therapy, as defined by IWCLL criteria
- Safety and toxicity
- Health-related quality of life (QoL)
- Cost-effectiveness

### Trial design

The trial is a phase III, multicentre, randomised, controlled, open, parallel-group trial in patients with previously untreated CLL. A total of 754 participants will be randomised on a 1:1 basis to receive therapy with either FCR or IR. Participants randomised to FCR will receive a maximum of six treatment cycles with each cycle being repeated every 28 days. Participants randomised to receive IR will receive six cycles of rituximab with each cycle being received every 28 days. Ibrutinib will be taken daily for 6 years or until MRD negativity has been recorded over a period of 6 months or until disease progression, whichever occurs first. Due to the different treatment modalities it is not possible to blind patients or clinicians to treatment allocation.

### Trial population

The main inclusion criteria are: being between 18 and 75 years old; B-CLL with a characteristic immunophenotype (including small lymphocytic lymphoma); Binet’s stages B, C or progressive stage A; require therapy by IWCLL criteria; able to provide written informed consent; considered fit for treatment with FCR as determined by the treating clinician; and ≤ 20% p53 deletion determined by fluorescent in situ hybridisation (FISH) (full inclusion and exclusion criteria listed in Table 1).

**Table 1:** Inclusion and exclusion criteria

Inclusion criteria	Exclusion criteria
• At least 18 years old • Maximum age of 75 years old • B-CLL with a characteristic immunophenotype, including small lymphocytic lymphoma • Binet’s stages B, C, or progressive stage A • Requiring therapy by the IWCLL criteria in that participants must have at least one of the following: ○ Evidence of progressive marrow failure as manifested by the development of, or worsening of, anaemia and/or thrombocytopenia ○ Massive (i.e. 6 cm below the left costal margin) or progressive or symptomatic splenomegaly ○ Massive nodes (i.e. 10 cm in longest diameter) or progressive or symptomatic lymphadenopathy ○ Progressive lymphocytosis with an increase of more than 50% over a 2-month period or lymphocyte doubling time (LDT) of less than 6 months as long as the lymphocyte count is over 30 × 10^9^/L ○ A minimum of any one of the following disease-related symptoms must be present: ▪ Unintentional weight loss more than or equal to 10% within the previous 6 months ▪ Significant fatigue (i.e. Eastern Cooperative Oncology Group performance status (PS) 2 or worse; cannot work or unable to perform usual activities) ▪ Fevers of greater than 38.0 °C for 2 or more weeks without other evidence of infection ▪ Night sweats for more than 1 month without evidence of infection • Considered fit for treatment with FCR as determined by the treating clinician • World Health Organisation (WHO) PS of 0, 1 or 2 • Able to provide written informed consent • Biochemical values must be within the following limits within 14 days prior to randomisation and at baseline: ○ Alanine aminotransferase (ALT) ≤ 3 × upper limit of normal (ULN). Aspartate aminotransferase (AST) ≤ 3 × ULN ○ Total bilirubin ≤ 1.5 × ULN, unless bilirubin rise is due to Gilbert’s syndrome or of non-hepatic origin	• Prior therapy for CLL • History or current evidence of Richter’s transformation • Major surgery within 4 weeks prior to randomisation • Active infection • >20% p53 deletion, determined by FISH • Past history of anaphylaxis following exposure to rat or mouse-derived CDR-grafted humanised monoclonal antibodies • Concomitant warfarin (or equivalent vitamin K inhibitor) or other orally administered anticoagulant treatment (anyone requiring anticoagulation treatment for more than 6 months is not eligible for trial entry. It is acceptable to be treated with LMW-heparin for < 6 months on the trial but other anticoagulation should be discussed with the CI) • Pregnancy, lactation or women of child-bearing potential unwilling to use medically approved contraception whilst receiving treatment and for 12 months after treatment with rituximab has finished, or 30 days after treatment with ibrutinib has finished, whichever is the latest. Women must agree to not donate eggs (ova, oocytes) for the purposes of assisted reproduction • Men whose partners are capable of having children but who are not willing to use appropriate medically approved contraception whilst receiving treatment and for 12 months after treatment with rituximab has finished, or 3 months after treatment with ibrutinib has finished, whichever is the latest, unless they are surgically sterile • CNS involvement with CLL • Symptomatic cardiac failure not controlled by therapy, or unstable angina not adequately controlled by current therapy (in patients with a significant cardiac history the left ventricular function should be assessed and patients with severe impairment should be excluded) • Respiratory impairment (bronchiectasis or moderate COPD) • Other severe, concurrent diseases or mental disorders that could interfere with participant’s ability to participate in the study • Inability to swallow orally administered medication • Disease significantly affecting gastrointestinal function and/or inhibiting small intestinal absorption (malabsorption syndrome, resection of the small bowel, poorly controlled inflammatory bowel disease, etc.) • Known HIV positive • Positive serology for hepatitis B (HB) defined as a positive test for HBsAg. In addition, if negative for HBsAg but HBcAb positive (regardless of HBsAb status), a HB DNA test will be performed and if positive the subject will be excluded (anyone who is HBsAg − ve/HBcAb + ve/HB DNA–ve should be referred to a liver disease specialist before the start of treatment with rituximab. During treatment, participants should be monitored and managed to prevent HBV reactivation) • Positive serology for hepatitis C (HC) defined as a positive test for HCAb, in which case reflexively perform a HC RIBA immunoblot assay on the same sample to confirm the result • History of prior malignancy, with the exception of the following: ○ Malignancy treated with curative intent and with no evidence of active disease present for more than 3 years prior to screening and felt to be at low risk for recurrence by treating physician ○ Adequately treated non-melanomatous skin cancer or lentigo maligna melanoma without current evidence of disease ○ Adequately treated cervical carcinoma in situ without current evidence of disease • Persisting severe pancytopenia (neutrophils < 0.5 × 10^9^/L or platelets < 50 × 10^9^/L) unless due to direct marrow infiltration by CLL • Current treatment with prednisolone of > 10 mg/day • Active haemolysis (patients with haemolysis controlled with prednisolone at a dose of 10 mg or less per day can be entered into the trial) • Patients with a creatinine clearance of less than 30 mL/min (either measured or derived by the Cockcroft Gault formula or alternative locally approved formula) • History of stroke or intracranial haemorrhage within 6 months prior to enrolment • Requirement for treatment with a strong CYP3A4/5 inhibitoror inducer • Cardiac event (e.g. recent myocardial infarction, coronary artery stent) requiring dual antiplatelet treatment

*CDR* complementarity-determining regions, *CI* chief investigator, *CLL* chronic lymphocytic leukaemia, *CNS* central nervous system, *COPD* chronic obstructive pulmonary disease, *DNA* deoxyribonucleic acid, *RIBA* Recombinant Immunoblot Assay, *FISH* fluorescent in situ hybridisation, *IWCLL* International Workshop on CLL, *LMW* low molecular weight

### Sample size

The trial is powered for the primary endpoint of PFS. The German CLL8 [7] trial showed a median PFS rate of 4.5 years in the FCR arm. To assess a superiority hazard ratio of 0.75 (that is, an improvement in median PFS to 6 years) with an overall 5% level of significance and 80% power, assuming a 4-year recruitment and a 4-year follow-up period, 355 participants are required per arm (710 participants overall). Accounting for a 5% dropout rate, which is in line with previous Leeds Institute of Clinical Trials Research (LICTR)-led trials in similar populations, 748 participants are required in order to observe 379 events.

A formal interim analysis on PFS will be carried out and reported to the Data Monitoring and Ethics Committee (DMEC) when half the required number of events have been observed. This is in order to allow any large differences between the randomisation arms to be reported early.

The O’Brien and Fleming alpha-spending function [8] will be used to adjust for multiple testing in order to conserve the overall type I error, which recommends that the interim results are compared to a *p* value of 0.005, and the final results are then compared to a *p* value of 0.048. In order to account for the interim analysis, the O’Brien and Fleming method recommends increasing the maximum required sample size and number of events by a factor of 1.008. Therefore, 754 participants will be recruited to the FLAIR trial in order to observe 382 events (191 for the interim analysis).

In order to recruit 754 participants over a 4-year period, the recruitment target is seven patients per month for the first 6 months whilst centres are opening to recruitment and 17 patients per month thereafter.

### Recruitment and consent

Participants will be recruited from multiple research centres from around the UK. Research centres will be required to have obtained ethical and management approvals and undertake a site initiation meeting with LICTR prior to the start of recruitment into the trial.

Potential participants will be identified by the clinical team at participating centres based on their diagnosis of CLL, and will be provided with verbal and written details about the trial. Informed consent will be collected by principal investigators at the participating hospitals, after a recommended minimum of 24 h to read and digest the information provided.

### Randomisation

Following consent, registration and confirmation of eligibility (Table 1), participants will be randomised into the trial by an authorised member of staff at the trial research site. Randomisation will be performed centrally using LICTR automated 24-h telephone randomisation system.

Participants will be randomised on a 1:1 basis to receive either FCR or IR and will be allocated a trial number. A computer-generated minimisation programme that incorporates a random element will be used to ensure that trial arms are well-balanced for the following participant characteristics: Binet’s stage (B, C or progressive stage A), age (≤65 years, > 65 years), gender (male, female), and randomising centre.

### Data collection and data monitoring

Data will be collected on paper Case Report Forms and entered into a validated trial database by LICTR. A validation check programme will be incorporated into the trial database to verify the data, and discrepancy reports will be generated for resolution by the investigator. Priority validations will be incorporated into the validation programme to ensure that any discrepancies related to participant rights, or safety, are expedited to sites for resolution. Data will be monitored for quality and completeness by LICTR. Missing data will be chased until received, confirmed as not available or the trial is at analysis. LICTR/the sponsor will reserve the right to intermittently conduct source data verification exercises on a sample of participants, which will be carried out by staff from LICTR/the sponsor. Source data verification will involve direct access to participant notes at the participating hospital sites and the central collection of copies of Consent Forms and other relevant investigation reports. Data will be held on a secure server at the University of Leeds and paper Case Report Forms stored in a locked unit, both will be accessible only to authorised trial staff.

QoL and participant-completed health economic data will be collected for patients who have given consent, and will be completed by the participant.

### Baseline assessments

Baseline assessments to be performed within 4 weeks of the start of treatment include: complete medical history, complete physical examination, WHO performance status, local haematology and biochemistry, serology for hepatitis B and C, and presence and level of P53 deletion (within 12 months prior to randomisation).

Participants will also undergo a chest X-ray if required, a computed tomography (CT) scan performed within 8 weeks prior to start of treatment and an electrocardiogram (ECG). Assessment of disease and a pregnancy test (if the participant is a woman of child-bearing potential) must also be performed within 2 weeks prior to start of treatment. Blood and bone marrow samples will be required and sent to the Haematological Malignancy Diagnostic Service (HMDS) for flow cytometry (bone marrow samples are only required if participants have a lymphocyte count of below 10 × 10^9^/L). For those who have given consent; blood, bone marrow and saliva samples will be sent to the UK CLL Trials Biobank (see ‘Substudies’ section).

For those who have given consent to QoL questionnaires and health economic assessments, baseline assessments must be completed prior to the participant becoming aware of their randomisation allocation.

### Intervention

Following randomisation to receive FCR or IR; those participants who receive FCR will receive 24/mg/m^2^/day of fludarabine and 150 mg/m^2^/day of cyclophosphamide orally on days 1 to 5, in addition to 375 mg/m^2^ on day 1 of cycle 1 and 500 mg/m^2^ on day 1 of cycles 2 to 6 of intravenous rituximab. Cycles of FCR are repeated every 28 days for a total of six cycles.

Participants randomised to IR will receive 375 mg/m^2^ on day 1 of cycle 1 and 500 mg/m^2^ on day 1 of cycles 2 to 6 of intravenous rituximab, and 420 mg of orally administered ibrutinib starting a day prior to the first dose of rituximab, on day 0 of cycle 1. Cycles of rituximab are repeated every 28 days for a total of six cycles, and ibrutinib will be given daily for up to 6 years, until disease progression, or unacceptable toxicity. The duration of ibrutinib therapy in responding patients will be defined by the eradication of MRD. For participants receiving ibrutinib, MRD will be assessed every 6 months in the peripheral blood, and if MRD negative, repeated after 3 months. If the repeat peripheral blood is MRD negative then after a further 3 months the peripheral blood and bone marrow will be assessed for MRD. If both are MRD negative then the patient will receive the same amount of treatment again as it took for them to become MRD negative, then they will stop taking ibrutinib. Participants would then restart ibrutinib if they become MRD positive, MRD positivity confirmed over 6 months, if MRD positivity is confirmed within 6 years post randomisation.

Participants will be assessed for their suitability for treatment within 1 week prior to day 1 of each cycle of FCR or R (for participants randomised to IR), and 3 monthly for the duration of treatment for participants randomised to IR, according to standard clinical practice. For participants randomised to FCR, participants should be questioned regarding nausea and vomiting or diarrhoea occurring with the prior cycle of therapy and if this is present then the fludarabine and cyclophosphamide should be given via the intravenous route from the next cycle onwards due to concerns over drug absorption. Intravenously administered fludarabine (25 mg/m^2^/day on days 1 to 3) and cyclophosphamide (250 mg/m^2^/day on days 1 to 3) regimens are recommended if the orally administered regimen is not tolerated. Patients receiving FCR will receive prophylaxis against *Pneumocystis jirovecii* pneumonia (co-trimoxazole or equivalent) and against herpes virus reactivation (acyclovir). G-CSF (as per standard dosing) for days 7 to 13 is recommended for all subsequent cycles of rituximab for participants who have had to have a previous dose delay due to neutropenia.

Participants can be withdrawn from treatment at the discretion of the treating clinician, and participants are free to withdraw consent from treatment or further follow-up at any time during the trial, without giving a reason, and without future treatment and care being affected in any way.

### Follow-up assessments

**Fig. 1:**
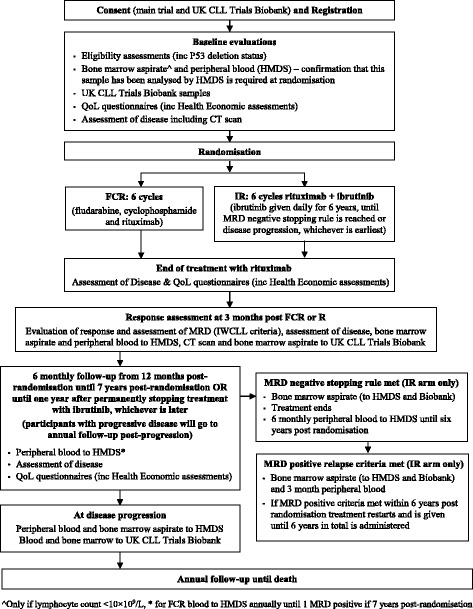
Trial flow diagram

Participants will be followed up as described in the trial flow diagram (Fig. 1). Participants will be followed up at 6 monthly intervals from 12 months post randomisation until 7 years post randomisation, withdrawal, disease progression requiring treatment, or death, whichever is earliest. Following 6-monthly follow-up, participants will be followed up annually until death or withdrawal, whichever is earliest.

**Fig. 2:**
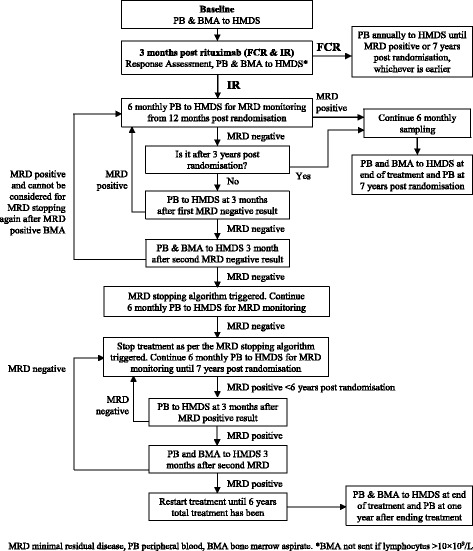
Minimal residual disease (MRD) sampling

**Fig. 3:**
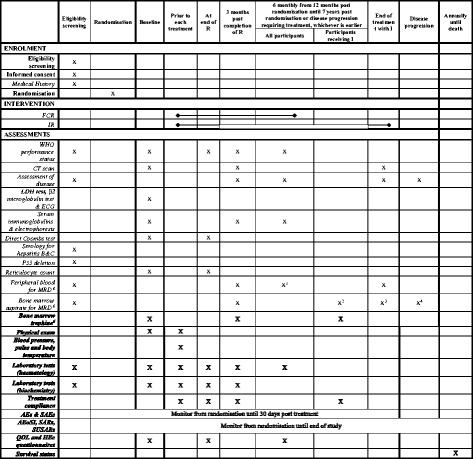
Standard Protocol Items: Recommendations for Interventional Trials (SPIRIT) Figure

Participants will be evaluated for response to treatment and assessment of MRD via bone marrow aspirate, according to the IWCLL response criteria, 3 months after the end of FCR or R (for participants randomised to IR) and again at the end of ibrutinib for participants randomised to IR. MRD will be assessed throughout the trial according to Fig. 2. A populated Standard Protocol Items: Recommendations for Interventional Trials (SPIRIT) figure is also provided in Fig. 3.

QoL and health economics questionnaires will be completed at the end of FCR or R, and then 6 monthly from 12 months post randomisation for 7 years or until disease progression, whichever is earliest.

Safety and toxicity data will be assessed continuously throughout the duration of the trial, as graded by the Common Terminology Criteria for Adverse Events (CTCAE) (v4.03) [9]. Events will be categorised as: suspected unexpected serious adverse reactions (SUSARs); serious adverse reactions SARs); serious adverse events (SAEs); adverse events of special interest (AEoSIs); and adverse events (AEs). AEoSIs are defined as: a major haemorrhage (resulting in: intraocular bleeding causing loss of vision; the need for a transfusion of two or more units of red cells or an equivalent amount of whole blood; hospitalisation; or the prolongation of hospitalisation); or an intracranial haemorrhage. Participants experiencing SAEs and AEs will be assessed from randomisation until 30 days after their last dose of trial treatment. Participants experiencing SARs, SUSARs and treatment-related deaths will be assessed from randomisation throughout the duration of the trial.

### Statistical methods and analysis

Statistical analysis is the responsibility of LICTR statisticians, and a final statistical analysis plan will be written and signed off before any analysis takes place. Health economic analyses will be carried out by a designated health economist at the University of Leeds, and a separate analysis plan will be written and signed off before any analysis takes place.

All analyses will be conducted on the intention-to-treat (ITT) population, where participants will be included according to the treatment that they were randomised to. A per-protocol (PP) analysis, where participants will be included according to the treatment they received, will be considered for the primary endpoint if there are a considerable number of protocol violators. The safety population will consist of all participants who receive at least one dose of treatment, where participants will be included according to the treatment that they received.

### Primary endpoint analysis

The primary endpoint of PFS will be assessed once 382 events have been observed overall (i.e. 382 participants have progressed/died). A formal interim analysis will be carried out when half the number of events (191 events) have been observed overall. Participants not having progressed or died at the time of the analyses will be censored at the last date they were known to be alive and progression-free. The analysis of primacy will use a Cox proportional hazards model [10] to compare the trial arms, adjusting for the minimisation factors excluding centre, to calculate the hazard ratio and associated 95% confidence intervals (CIs). The *p* value will be compared to 0.048 to account for the formal interim analysis. In addition, median survival estimates and Kaplan-Meier curves [11] will be produced by trial arm.

### Secondary endpoint analyses

Overall survival will be assessed at the same time as the primary endpoint using a Cox proportional hazards model to compare the trial arms, adjusting for the minimisation factors excluding centre. Kaplan-Meier curves, 95% CIs and median survival estimates will be produced by trial arm and overall.

The proportion of participants with undetectable MRD (i.e. MRD negative) will be summarised by trial arm and overall at each time point that MRD is assessed. A binary logistic regression model will be used to compare trial arms in terms of the proportion of participants who become MRD negative at any stage during the trial, adjusting for the minimisation factors excluding centre. Time to participants achieving MRD negativity will also be assessed in the IR arm alone.

Binary logistic regression models will be used to compare trial arms in terms of the proportion of participants who achieve a Complete Response (CR or CRi) and who achieve an Overall Response (at least a PR) at any stage during the trial, adjusting for the minimisation factors excluding centre.

Occurrence and frequency of SUSARs, SARs, SAEs, AEoSIs, AEs and treatment-related deaths will summarised by trial arm and overall. In addition, all events will be summarised by maximum CTCAE grade reported and seriousness criteria; and all serious events will be summarised by MedDRA System Organ Class, by trial arm and overall.

Mean quality of life (QoL) scores and 95% CIs, adjusted for the baseline score, will be calculated for all domains of the European Organisation for Research and Treatment of Cancer (EORTC) QLQ-C30 and CLL-specific module EORTC QLQ-CLL16 for each trial arm and at each assessment time point and overall. Wilcoxon’s rank sum tests will be used to compare the change in score for the trial arms from baseline to each time point.

A cost-effectiveness analysis, incorporating the development of a decision analytic cost effectiveness Markov/semi-Markov state model, using quality-adjusted life years (QALYs) estimated from utility weights and health state scores, will be carried out using the euroQol five dimensions (EQ-5D) and 12-item Short Form Health Survey (SF12) collected at baseline, end of FCR/R and 6 monthly from 12 months post randomisation.

Pre-specified exploratory analyses will be conducted for subgroups of interest as appropriate, but will be interpreted with caution and treated as hypothesis-generating.

### Interim reports

An independent DMEC will review the safety and ethics of the trial whilst participants are receiving trial treatment; reviewing unblinded safety updates at 3-monthly intervals, and more detailed unblinded reports annually.

A formal interim analysis on the primary endpoint of PFS will be reviewed by the DMEC when half of the required number of events have been observed (191 over FCR and IR combined). A Cox proportional hazards model will be used to compare trial arms in terms of PFS, adjusting for the minimisation factors excluding centre, where the result will be compared to a *p* value of 0.005.

The DMEC, in light of the interim data, and of any advice or evidence they wish to request, will advise the TSC if there are any concerns or reasons that the trial should not continue, or if they believe that the trial results should be shared further.

### Trial organisation and administration

The trial was developed by the FLAIR Trial Management Group (TMG), with the support of the UKCLL/NCRI CLL Clinical Trials Subgroup. The trial is sponsored by the University of Leeds ((R&D Office, 34 Hyde Terrace, Leeds, LS2 9LN); sponsor reference: HM13/10747)), run and co-ordinated by LICTR, and is registered (ISRCTN01844152, EudraCT Number 2013-001944-76). The trial will be conducted in accordance with the principles of Good Clinical Practice (GCP) in clinical trials, as applicable under UK regulations, the NHS Research Governance Framework and through adherence to LICTR standard operating procedures (SOPs). LICTR and the sponsor have systems in place to ensure that serious breaches of GCP of the trial protocol are identified and reported. A sample of data will be assessed by on-site source data verification by LICTR and this is not independent from the sponsor. The University of Leeds will be liable for negligent harm caused by the design of the trial. No additional compensation for clinical negligence will be provided for trial participants over that which is available to NHS patients. Ibrutinib was only provided by the sponsor for the duration of protocol treatment and subsequent treatment will be as per standard care.

A core Internal Project Team (IPT), a TMG, a TSC, and a DMEC will be established. The independent DMEC will review the safety and ethics of the trial, as overseen by the independent TSC. The TSC will monitor trial progress and the overall direction. The results of the study will be published in peer-reviewed publications and will be presented at relevant national and international conferences. There are no plans to use professional writers for presenting the outcomes of this trial. Any protocol changes are disseminated by LICTR to the relevant parties. A Standard Protocol Items: Recommendations for Interventional Trials (SPIRIT) Checklist has been prepared for this manuscript (Additional file 1).

### Protocol amendments

The trial opened to recruitment in August 2014 using protocol version 1.0 (6 March 2014).

The protocol was amended to version 2 on 4 September 2014 to include the additions of long-term orally administered anticoagulant use and cardiac events requiring dual antiplatelet therapy to the exclusion criteria on safety grounds. This change was implemented before any participants were randomised.

The protocol was amended to version 3.0 on 29 September 2016 to modify the MRD stopping criteria based on updated external MRD data. A criteria for restarting treatment based on MRD relapse was also added at this point. Secondary endpoints relating to pattern of MRD relapse, retreatment and MRD response over time in participants randomised to IR were also added. These changes were implemented before any participants had reached either the original or the amended MRD negative stopping criteria.

Both amendments were reviewed and approved by the sponsor, the REC and MHRA.

### Substudies

Trial participants will also be invited to take part in the UK CLL Trials Biobank at the University of Liverpool. Participants are approached at baseline with a separate consent form (REC reference 14/NW/1014) and biological samples are sent to the University of Liverpool.

## Discussion

The FLAIR trial has been designed in order to assess the effectiveness of combination therapy with ibrutinib plus rituximab in terms of PFS in patients with newly diagnosed CLL, compared to the current standard of fludarabine, cyclophosphamide and rituximab.

There has been considerable enthusiasm for the trial amongst both clinicians and patients. Current treatments for CLL are moving away from standard chemotherapy regimens given over 6 months, towards longer-term treatment with more targeted small molecules. Treatment with ibrutinib is attractive to patients due to its oral administration and apparent low toxicity profile compared with FCR. In England, ibrutinib is currently only available through clinical trials in the front-line treatment setting for patients without the 17p deletion, and thus recruitment into the trial has been positive and above target.

However, the superiority of ibrutinib over the most effective chemoimmunotherapy (FCR) has yet to been demonstrated and the long-term effects of ibrutinib have yet to be fully determined. This trial will follow up participants until death to understand the durability of any response over FCR.

Novel, small molecules, such as ibrutinib, have a significant financial cost compared with FCR and so it is vital to collect health economic data. Ibrutinib is currently licensed for continuous use until disease progression or unacceptable toxicity. This trial will evaluate a disease-based stopping rule using minimal residual disease where ibrutinib treatment will continue until disease is no longer detectable, the first time that this has been looked at in the front-line setting.

### Trial status

The FLAIR trial opened to recruitment in August 2014 using protocol version 1.0 (6 March 2014) and is currently recruiting above target. As of 24 February 2017, 537 participants have been randomised. Recruitment is due to close in August 2018.

## Additional files

Additional file 1: SPIRIT Checklist. (DOCX 48 kb)

Additional file 2: List of centres. (DOCX 18 kb)

## Abbreviations

AE: Adverse event
ALT: Alanine aminotransferase
AST: Aspartate aminotransferase
BCR: B-cell receptor
BTK: Bruton’s tyrosine kinase
CI: Confidence interval
CLL: Chronic lymphocytic leukaemia
CR: Complete response
CRF: Case Record Form
CRi: Complete response with incomplete marrow recovery
DMEC: Data Monitoring and Ethics Committee
ECG: Electrocardiogram
EMA: European Medicines Agency
EORTC: European Organisation for Research and Treatment of Cancer
FCR: Fludarabine, cyclophosphamide and rituximab
FISH: Fluorescent in situ hybridisation
GCP: Good Clinical Practice
HIV: Human immunodeficiency virus
IR: Ibrutinib and rituximab
ITT: Intention-to-treat
IV: Intravenous
IWCLL: International Workshop on Chronic Lymphocytic Leukaemia
LICTR: Leeds Institute of Clinical Trials Research
MRD: Minimal residual disease
NCRI: National Cancer Research Institute
NHS: National Health Service
NICE: National Institute for Health and Clinical Excellence
NRES: National Research Ethics Service
OS: Overall survival
PFS: Progression-free survival
PS: Performance status
QoL: Quality of life
REC: Research Ethics Committee
SAE: Serious adverse event
SLL: Small lymphocytic lymphoma
SOP: Standard operating procedure
SUSAR: Suspected unexpected serious adverse reaction
TMG: Trial Management Group
TSC: Trial Steering Committee
WHO: World Health Organisation
