# Supplementary material for: Assessment of ibrutinib plus rituximab in front-line CLL (FLAIR trial): study protocol for a phase III randomised controlled trial
Source: Trials. 2017 Aug 22;18:387. doi: 10.1186/s13063-017-2138-6 (PMC5568356; doi:10.1186/s13063-017-2138-6)
Supplement: Supplementary file 2 — List of centres. (DOCX 18 kb) [file 13063_2017_2138_MOESM2_ESM.docx]

**Additional File 2. FLAIR centres open to recruitment on 08/02/17.**

| **Centre** |
| --- |
| King's College Hospital |
| Nottingham University Hospital |
| Churchill Hospital |
| Christie Hospital |
| Royal Hallamshire Hospital |
| Southampton General Hospital |
| Worcestershire Acute Hospitals NHS Trust |
| University Hospital of Wales, Cardiff |
| Kent and Canterbury Hospital |
| Western General Hospital |
| St James's University Hospital |
| Russells Hall Hospital |
| Castle Hill Hospital |
| Royal Bournemouth General Hospital |
| Leicester Royal Infirmary |
| University College London Hospital |
| Good Hope Hospital |
| Royal Cornwall Hospital |
| Aberdeen Royal Infirmary |
| Blackpool Victoria Hospital |
| Worthing Hospital |
| Colchester General Hospital |
| Birmingham Heartlands Hospital |
| Milton Keynes Hospital |
| Buckinghamshire Healthcare NHS Trust |
| Salisbury District Hospital |
| Southmead Hospital |
| Epsom and St Helier Hospital |
| Singleton Hospital |
| Addenbrooke's Hospital |
| Bradford Royal Infirmary |
| Musgrove Park Hospital |
| Royal Marsden Hospital |
| QE Hospital Birmingham |
| Manchester Royal Infirmary |
| Doncaster Royal Infirmary |
| Rotherham General |
| Torbay District General Hospital |
| Hammersmith Hospital |
| Glan Clwyd Hospital |
| Royal Devon and Exeter Hospital |
| QE Hospital Gateshead |
| Grantham and District General Hospital |
| Lincoln County Hospital |
| Pilgrim Hospital Boston |
| Royal Surrey County Hospital |
| Royal Derby Hospital |
| Bristol Haematology and Oncology Centre |
| Ysbyty Gwynedd |
| Raigmore Hospital |
| Wrexham Maelor Hospital |
| Queen Margaret Hospital, Dunfermline |
| Craigavon Area Hospital |
| South Tees NHS Foundation Trust |
| Northampton General Hospital |
| Royal Albert Edward Infirmary |
| Scunthorpe General Hospital |
| York Hospital |
| Huddersfield Royal Infirmary |
| Sandwell General Hospital |
| Poole Hospital |
| Gloucestershire Royal Hospital |
| University Hospitals of North Midlands NHS Trust |
| Scarborough Hospital |
| Beatson Oncology Centre |
| Princess Royal University Hospital |
| NHS Lanarkshire |
| Belfast City Hospital |
| Pennine Acute Hospitals NHS Trust |
| Glangwili General Hospital |
| Calderdale Royal Hospital |
| Harrogate District Hospital |
| Hampshire Hospitals NHS Foundation Trust |
| Croydon University Hospital |
| George Elliot Hospital |
| St Richard's Hospital |
| Basildon Hospital |
| Surrey and Sussex Healthcare NHS Trust |
| Salford Royal Hospital |
| Royal Alexandra Hospital Paisley |
| Altnagelvin Hospital |
| Queen's Hospital Romford |
| Diana Princess of Wales Hospital |
| University Hospital Aintree |
| King's Mill Hospital |
| Queen Alexandra, Portsmouth |
| St Bartholomew's Hospital |
| Ipswich Hospital |
| University Hospital South Manchester |
| Great Western Hospital |
| Victoria Hospital, Kirkcaldy |
| Borders General Hospital |
| University Hospital Coventry |
| New Victoria Hospital Glasgow |
| Cheltenham General Hospital |
| Royal Liverpool Hospital |
| Barnet/Chase Farm Hospitals |
| Withybush General Hospital |
| Royal Gwent Hospital |
| Nevill Hall Hospital |
| Peterborough City Hospital |
